# Zeamide, a Glycosylinositol Phosphorylceramide with the Novel Core Ara*p*(1β→6)Ins Motif from the Marine Sponge *Svenzea zeai*

**DOI:** 10.3390/molecules22091455

**Published:** 2017-09-01

**Authors:** Gerardo Della Sala, Roberta Teta, Germana Esposito, Joseph R. Pawlik, Alfonso Mangoni, Valeria Costantino

**Affiliations:** 1 The NeaNat Group, Dipartimento di Farmacia, Università degli Studi di Napoli Federico II, via D. Montesano 49, Napoli 80131, Italy; gerardo.dellasala@unina.it (G.D.S.); roberta.teta@unina.it (R.T.); germana.esposito@unina.it (G.E.); alfonso.mangoni@unina.it (A.M.); 2 Department of Biology and Marine Biology, University of North Carolina Wilmington, Center for Marine Science, 5600 Marvin K Moss Lane, Wilmington, NC 28409, USA; pawlikj@uncw.edu

**Keywords:** glycosylinositol phosphorylceramides (GIPCs), d-arabinose, microscale chemical degradation

## Abstract

Glycosylinositol phosphorylceramides (GIPCs) show a great structural diversity, but all share a small number of core structures, with a glucosamine, a mannose, or a glucuronic acid as the first sugar linked to the inositol. The Caribbean sponge *Svenzea zeai* was shown to consistently contain zeamide (**1**), the first example of a new class of GIPCs, in which the inositol is glycosylated by a d-arabinose. The structure of zeamide was determined by spectroscopic analysis (NMR, MS, ECD) and microscale chemical degradation. The 6-*O*-β-d-arabinopyranosyl-*myo*-inositol (d-Ara*p*(1β→6)Ins) core motif of zeamide is unprecedented not only among GIPCs, but also in any natural glycoconjugate.

## 1. Introduction

**Figure 1:**
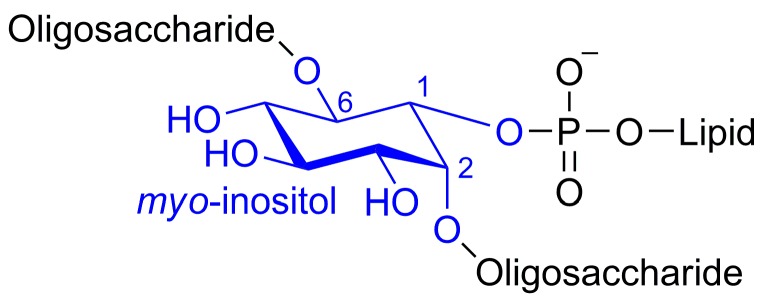
Core structure of a typical glycosylphosphatidylinositol (GPI). The inositol may be glycosylated at *O*-2, at *O*-6, or at both positions. The lipid is generally a diacylglycerol, but in glycosylinositol phosphorylceramides (GIPCs) the lipid is a ceramide.

Glycosylphosphatidylinositols (GPIs) are widespread in all kingdoms of living organisms. They are composed of a lipid moiety (most often, but not exclusively, a diacylglycerol) linked through a phosphodiester bond to a *myo*-inositol unit, which is in turn glycosylated at *O*-2, at *O*-6, or at both positions by oligosaccharide chains ranging from 1 to 10 sugar residues or, in some cases, by much longer glycans (Figure 1). The most important representatives of this class are the so-called GPI-anchors [1], which are used ubiquitously in eukaryotes to anchor proteins to the cell wall. The core oligosaccharide of GPIs consists, in sequence, of one glucosamine, three mannoses, and a terminal phosphoethanolamine, which is amide-bonded to the C-terminus of the protein. Proper biosynthesis of GPI-anchors is essential for the survival of the animals [2], plants [3], and protozoa [4] producing them.

GPIs based on a ceramide backbone (also called glycosylinositol phosphorylceramides, or GIPCs), are less widespread, but show a well-defined taxonomic distribution [5], and have been proposed as the major sphingolipids on earth [6]. GPI-anchors with a ceramide backbone are found in protozoa and fungi [7]. Protozoa such as *Trypanosoma cruzi* also contain large amounts of non-protein-linked GIPCs with oligosaccharide sequences similar or identical to those of GPIs [8]. Many species of fungi contain GIPCs with oligosaccharides mainly composed of mannose [9]. Higher plants contain large amounts of GIPCs with a glucuronic acid as the first sugar of the oligosaccharide chain [10,11]. In contrast, GIPCs have not been found in mammals. The possible role of GIPC in higher plants and other organisms has been recently reviewed [6].

Porifera, the sponges, are a rich source of glycolipids with unusual structures (Figure S1), including a whole new class of immunostimulating α-galactosylceramides [12], the *O*-prenylated glycosyl ceramides plakosides [13], the monohexofuranosyl ceramide ectyoceramide [14], glycoglycerolipids such as crasserides and isocrasserides [15] characterized by a unique five-membered cyclitol, terpene glycosides such as plakylosides [16], and atypical glycosides such as simplexide [17], the diglycoside of a very-long-chain secondary alcohol. However, no GIPCs (and, more generally, no GPIs) have been reported from the Porifera so far. The only inositol-containing glycolipid reported so far from sponges are discoside [18], a diacylated inositol α-mannoside which contains the mannosyl-*myo*-inositol core structure characteristic of GPI of fungi, mycobacteria [19], and plakohopanoid, which has the same core structure, but is acylated by a C_32_ hopanoid acid [20] (see Figure S1).

**Figure 2:**
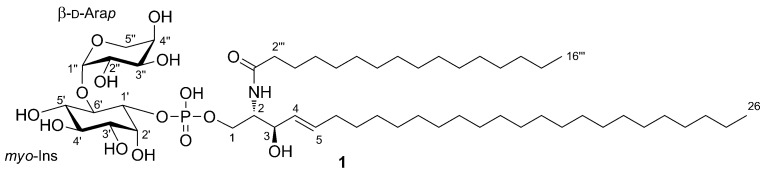
Structure of a representative homologue of zeamide (**1**).

We wish to report here the isolation and structure elucidation of zeamide (1) (Figure 2), a glycosylinositol phosphorylceramide showing the 6-*O*-β-d-arabinopyranosyl-*myo*-inositol (d-Ara*p*(1β→6)Ins) core motif, which is unprecedented not only among GIPCs, but also in any natural glycoconjugate.

## 2. Results and Discussion

A sample (410 g wet weight) of the sponge *Svenzea zeai* was collected along the coast of the Florida Keys in June 2016 at −15 m, immediately frozen and kept frozen until extraction, and extracted using our standard procedure [21] involving extraction with MeOH/CHCl_3_ mixtures and partitioning between H_2_O and BuOH. The organic extract was subsequently subjected to reversed- and normal-phase column chromatography, yielding a fraction mainly composed of phosphoglycosylceramides, which were further purified by crystallization from MeOH to give 9.0 mg of pure zeamide **1** as a mixture of homologues. As often occurs for glycolipids from marine sponges [22], the mixture could not be separated into chemically homogeneous compounds, but nonetheless was perfectly suitable for NMR studies. The ^1^H-NMR proton spectrum of **1** was clearly suggestive of a glycosphingolipid, in that it contained signals for a sphingosine double bond (δ 5.45, dd, *J* = 15.3 and 7.7 Hz, H-4; δ 5.71, ddd, *J* = 15.3, 6.9, and 6.9 Hz, H-5), an anomeric proton (δ 5.36, d, *J* = 3.7 Hz, H-1″), and the methyl groups of *n*-(δ 0.90, t, *J* = 7.1 Hz), *iso*-(δ 0.88, d, *J* = 6.7 Hz), and internally branched (δ 0.86, d, *J* = 6.8 Hz) alkyl chains (distributed among fatty acid chains and sphingosine chains). Integration of the methyl signals showed that the ratio of *n*-chains/iso-chains/internally branched chains was about 64:29:7.

The high-resolution ESI-MS (negative ion mode) of zeamide **1** showed a homologous series 85 of deprotonated molecular [M − H]^–^ ions at *m*/*z* 994.6561, 1008.6716, 1022.6874, 1036.7029, and 1050.7184 in accordance with the molecular formulas C_51_H_97_NO_15_P + *n* CH_2_ (*n* = 0–4, four unsaturations). Additionally, a careful examination of the spectrum revealed the presence of a second homologous series of deprotonated molecular [M − H]^−^ ions at *m*/*z* 996.6713, 1010.6866, and 1024.6998, in accordance with the molecular formulas 89 C_51_H_99_NO_15_P + *n* CH_2_ (*n* = 0–2, three unsaturations), originating from a series of less abundant homologues with a saturated sphingosine (see below). High-resolution ESI-MS (positive ion mode) contained a similar series of sodiated molecular [M − H + 2Na]^+^ ions, in accordance with the same molecular formulas; the doubly sodiated adduct suggested the presence of the strongly acidic phosphodiester function.

In spite of our attempts, it was not possible to obtain any pure homologue from this mixture, so all NMR studies were performed on the mixture, taking advantage of the fact that ^1^H and ^13^C signals of individual homologues were superimposable (except where noted in Table 1). Because of the many overlapped signals in the proton spectrum, a *z*-filtered TOCSY spectrum recorded with digital resolution of 0.94 Hz in the F2 dimension was used to generate the subspectra of individual spin systems (Figure S2) and to measure most proton-proton coupling constants (Table 1).

Analysis of the COSY and HSQC spectra allowed the assignment of all the signals of a dihydroxylated, monounsaturated sphingosine and of a fatty acid residue (Table 1), which were shown to be connected to each other through an amide bond to form a ceramide by the HMBC correlation between H-2 (δ 3.91) and the carbonyl carbon atom C-1′′′ (δ 175.9). The anomeric proton H-1′′ served as the starting point for the sequential assignment through the COSY and TOCSY spectra of protons of the only sugar residue present in the molecule (Table 1). It was revealed to be a pentose in the pyranose form, as demonstrated by the HMBC correlation between the anomeric proton and the oxymethylene carbon C-5′′ (δ 64.9). Coupling constants showed that H-2′′ and H-3′′ (sharing a 9.9 Hz coupling) were axial, and H-1′′ and H-4′′ were equatorial. Therefore, the sugar residue was identified as a β-arabinopyranoside.

The proton spectrum of compound **1** also contained signals for six oxymethine protons. The COSY spectrum showed them to be cyclically arranged, and therefore to be the protons of an inositol ring (Table 1). All the inositol ring protons showed large axial-axial mutual couplings and were therefore axial, except for H-2′ (δ 4.27, t, *J* = 2.7 Hz), which was equatorial. This indicated that the inositol was a *myo*-inositol. The *myo*-inositol was glycosylated by the arabinose through a (1→6) linkage, as shown by the HMBC correlations of H-1′′ with C-6′ and of H-6′ with C-1′′. Finally, the *myo*-inositol C-1′ and ceramide C-1 were linked through a phosphodiester bond, as suggested by the additional ^1^H-^31^P splitting of the methylene protons at C-1 and of H-1′. The presence of this phosphodiester bond was confirmed by recording a ^1^H-^31^P HMBC experiment: the spectrum contained, as the only correlation peaks, the ^1^H-^31^P correlations of H-1a, H-1b, and H-1′ with the phosphate P atom.

In addition to the signals described so far, the proton NMR of **1** also displayed some weaker signals, indicating the presence of a minor analogue. The amounts of this minor analogue were estimated as about 20% compared to the major analogue by the integration of the signal at δ 3.84 (H-2). Using this proton signal as a starting point, it was possible to use the TOCSY spectrum to identify all the signals of this spin system (Table 1), and to use the COSY spectrum to arrange them in a sequence. Overall, the signals were indicative of the presence of minor amounts of homologues with a saturated dihydroxylated sphingosine, in accordance with the MS data.

**Scheme 1:**
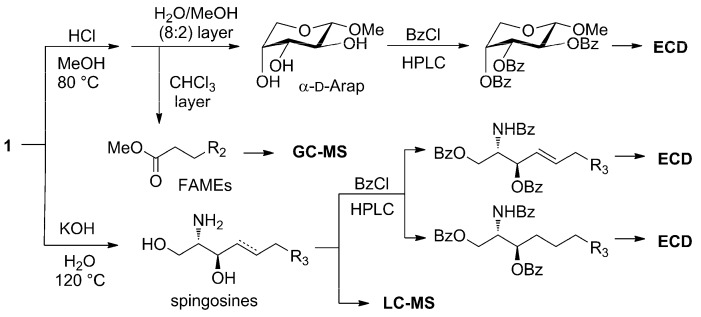
Microscale degradation procedure used to determine structural details of zeamide (**1**).

Microscale chemical degradation was needed to determine the absolute configuration of the arabinose residue and to elucidate the detailed structure of the ceramide part of the molecule (Scheme 1). A small amount (100 μg) of compound **1** was subjected to methanolysis with HCl in MeOH, and the reaction mixture was subsequently partitioned between H_2_O/MeOH (8:2) and CHCl_3_. The aqueous layer, containing methyl arabinosides, was benzoylated and purified by HPLC, yielding methyl tri-*O*-benzoyl-α-d-arabinopyranoside, which was identified by a comparison of its HPLC retention time and ECD (Electronic Circular Dichroism) spectrum with those of an authentic sample prepared from d-arabinose [23]. The organic phase, containing fatty acid methyl esters, was analyzed by GC-MS. The results, compiled in Table 2, showed a preponderance of *iso-*fatty acid residues and significant amounts of internally branched fatty acid residues, overall accounting for nearly all the branched alkyl chains revealed from the ^1^H-NMR spectrum. Therefore, the sphingosines were expected to be mostly unbranched.

It was not possible to isolate any sphingosine from the methanolysis products. Therefore, a further aliquot (200 μg) of compound **1** was subjected to basic hydrolysis with aqueous KOH at 100 °C for 72 h. The reaction mixture was analyzed by LC-MS, and shown to contain both unsaturated and saturated sphingosines ranging from C_24_ to C_28_; the C_26_ unsaturated sphingosine was by far the most abundant homologue (62.1%), followed by the C_26_ saturated sphingosine (Table 3). The retention times of the sphingosines varied regularly with the chain length, as expected for homologous unbranched sphingosines (Figure S3); only minor amounts of branched sphingosines, isomeric with unbranched sphingosine but with shorter retention times, were observed.

The relative and absolute configuration of the sphingosines was established by chiroptical methods. The reaction mixture from hydrolysis was benzoylated and purified by normal-phase HPLC, giving two fractions composed of, respectively, homologous tribenzoylated sphingosines and homologous tribenzoylated dihydrosphingosines. The ECD spectra of these fractions matched those reported for, respectively, tribenzoylated C_18_ d-*erythro*-sphingosine and tribenzoylated C_18_ d-*erythro*-dihydrosphingosine [24].

**Figure 3:**
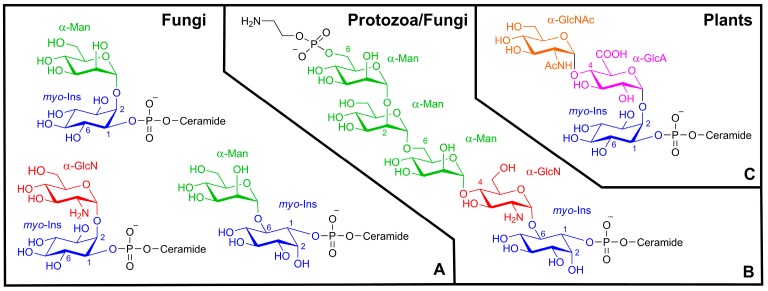
Core structures of known natural GIPCs. (**A**) The three core structures (Man(1α→2)*myo*-inositol-1-PO_4_-Cer, Man(1α→6)*myo*-inositol-1-PO_4_-Cer, and GlcN(1α→2)*myo*-inositol-1-PO_4_-Cer) of GIPCs found in fungi; (**B**) The core structure (ethanolamine-PO_4_-6-Man(1α→2)Man(1α→6)Man(1α→4)GlcN(1α→6)*myo*-inositol-1-PO_4_-Cer) of GPI-anchors based on a ceramide lipid moiety found in fungi and protozoa; (**C**) The core structure (GlcNAc(1α→4)GlcA(1α→2)*myo*-inositol-1-PO_4_-Cer) of GIPCs found in plants. Abbreviations: Ins: inositol; Glc: glucose; GlcN: 2-amino-2-deoxyglucose (glucosamine); GlcNAc: 2-acetamido-2-deoxyglucose (*N*-acetylglucosamine); GlcA: glucuronic acid; Man: mannose.

Although GIPCs show great structural diversity, they all share a small number of core structures, with a glucosamine, a mannose, or a glucuronic acid as the first sugar linked to the inositol (Figure 3). Zeamide (**1**) is the first example of a new class of GIPCs, in which the inositol is glycosylated by a d-arabinose.

It is worth noting that zeamide **1** is not a trace metabolite in *S. zeai*, a species widespread in the Caribbean. Although the isolation procedure was far from being quantitative, still 9 mg of **1** could be obtained from 410 g of wet weight of sponge. In addition, the analysis of two further specimens of *S. zeai*, collected in different years and locations, gave similar results as for zeamide content, showing that zeamide is consistently present in *S. zeai*. Marine sponges are filter-feeding organisms, and many of them (the so-called high-microbial abundance (HMA) sponges, which include *S. zeai* [25]) contain large amounts of symbiotic microorganisms, so the actual biosynthetic origin of a metabolite found in a marine sponge is often difficult to determine [26,27].

A hint of the biosynthetic origin of GIPCs is usually given by the nature of the ceramide. GIPCs from plants usually contain trihydroxylated, saturated sphinganines (phytosphingosines), and 2-hydroxy fatty acids; fungal GIPCs are often characterized by polyunsaturated and methyl-branched sphingosines, and very long (C_24_ or C_26_) fatty acids. The ceramide of zeamide does not fit either of these schemes. It is composed of very long-chain (C_24_-C_28_) d-*erythro*-sphingosines (which are very rare in all kinds of living organisms, including sponges) and a high proportion of branched saturated fatty acids. On the other hand, branched fatty acids are very common in lipids from marine sponges and are considered to be produced by bacterial symbionts. The metabiome of HMA sponges has revealed the widespread occurrence of sponge-specific bacterial polyketide synthases, named SupA and Swf, which have been predicted to synthesize methyl-branched fatty acids [28,29]. However, any experimental evidence of the bacterial production of methyl-branched fatty acids has been reported to date from this sponge species.

Taken together, these data suggest that zeamide is either a product of the biosynthetic metabolism of the sponge, or the product of some symbiotic microorganisms that are very specific to this sponge species.

## 3. Materials and Methods

### 3.1. General Experimental Procedure

Optical rotations were measured using a Jasco P-2000 polarimeter at the sodium D line. ECD spectra were recorded using a Jasco-715 (Jasco Europe, Carpi, Italy) spectropolarimeter. NMR spectra were determined on Varian Unity Inova (Varian ,Palo Alto, CA, USA)pectrometers at 700 MHz and 500 MHz; chemical shifts were referenced to the residual solvent signal (CD_3_OD: δ_H_ 3.31, δ_C_ 49.30). For an accurate measurement of the coupling constants, the one-dimensional ^1^H-NMR spectra were transformed at 64-K points (digital resolution: 0.09 Hz). The HSQC spectra were optimized for ^1^*J*_CH_ = 142 Hz; the ^13^C and ^31^P HMBC experiments for ^2,3^*J*_CH_ = 8.3 Hz and ^3^*J*_PH_ = 8 Hz, respectively. High-resolution ESI-MS and HR-ESI-HPLC experiments were performed on a Thermo LTQ Orbitrap XL mass spectrometer coupled to a Thermo U3000 HPLC system. Fatty acid methyl esters (FAMEs) were analyzed by GC/MS (Agilent, Cernusco sul Naviglio (Mi), Italy, 6850 series II/5973 Network MSD) on an HP-5MS capillary column (Agilent, 5% Phenyl Methyl Siloxane) (30 m, 0.25 mm φ, 0.25 μm). Helium was used as a carrier gas, injection was in split mode, and the program was as follows: hold 150 °C for 15 min, heat to 300 °C with 5 °C/min, hold 300 °C for 10 min. High performance liquid chromatography (HPLC) separations were achieved on an Agilent 1260 Infinity Quaternary LC apparatus equipped with a Diode-Array Detector (DAD).

### 3.2. Collection, Extraction, and Isolation

A specimen of *Svenzea zeai* (410 g wet weight) was collected in July 2016 by scuba diving at depths of 15 m offshore the Florida Keys (USA). The sample was a relatively small portion of a much larger sponge, and was excised with a sharp scalpel to minimally affect the remaining sponge tissue and allow recovery and regrowth. After collection, the sample was unambiguously identified onboard using a web-based photographic and taxonomic key, The Sponge Guide (www.spongeguide.org), with subsequent confirmation by sponge taxonomist Dr. Sven Zea. The sample was frozen immediately after collection and stored at −20 °C until extraction. A voucher specimen of the organism is stored at Dipartimento di Farmacia, Università degli Studi di Napoli “Federico II” with the reference number 2316. The sponge (410 g wet weight, 600 mL volume) was cut into pieces and extracted with MeOH (4 × 2.5 L), MeOH/CHCl_3_ (3 × 2.5 L) and CHCl_3_ (2 × 2.5 L). The MeOH extract was partitioned with H_2_O and BuOH; the organic layer was added to the CHCl_3_ extracts, affording 5.7 g extract, which was chromatographed on a column packed with RP-18 silica-gel. The fraction eluted with MeOH/CHCl_3_ (9:1, 600 mg) was chromatographed on a silica normal-phase column; a fraction eluted with MeOH (62 mg) was partitioned in a two-phase system CHCl_3_/MeOH/H_2_O (8:4:3); the upper aqueous layer (33 mg) was dried, and the residue precipitated from MeOH to give a white solid (9 mg) composed of pure zeamide (**1**) as a mixture of homologues. Analysis of additional samples of *S. zeai* gave similar results. A specimen collected in the Bahamas in 2004 along the coasts of San Salvador Island (reference No. 112004, 450 g wet weigh) yielded 8.5 mg of zeamide (**1**), and a specimen collected in 2012 in Sweeting Cay (Grand Bahamas) (reference No. 1210 bis, 430 g wet weight) yielded 9.4 mg of zeamide (**1**).

### 3.3. Zeamide (***1***)

White amorphous solid, ${\left[\alpha \right]}_{D}^{25}$ = −10.6 (MeOH); HRESIMS (negative ion mode, MeOH) *m*/*z* 994.6561, 1008.6716, 1022.6874, 1036.7029, 1050.7184 [M − H]^−^ (calcd for C_51_H_97_NO_15_P + *n* CH_2_, *n* = 0–4, 994.6601, 1008.6758, 1022.6914, 1036.7071, 1050.7228) and *m*/*z* 996.6713, 1010.6866, 1024.6998 (calcd. for C_51_H_99_NO_15_P + *n* CH_2_, *n* = 0–2, 996.6758, 1010.6914, 1024.7071); ^1^H and ^13^C-NMR: Table 1.

### 3.4. Methanolysis of Zeamide (***1***)

Zeamide (100 μg) was dissolved in 1 M HCl in 91% MeOH (1 mL) in a sealed tube and kept at 80 °C for 12 h. The reaction product was dried under nitrogen, and then partitioned between a mixture of H_2_O/MeOH (8:2) and CHCl_3_. The organic layer was analyzed by GC-MS for fatty acid methyl esters, which were identified by a comparison of their retention times and mass spectra with those of authentic samples. The results are compiled in Table 2.

The aqueous layer was concentrated under vacuum, and the residue was benzoylated with benzoyl chloride (50 μL) and pyridine (500 μL) at 25 °C for 16 h. The reaction was quenched with MeOH, and after 1 h the mixture was dried under nitrogen. Methyl benzoate was removed by keeping the residue under high vacuum with a freeze dryer for 72 h. The residue was purified by HPLC (column: Phenomenex Silica (2), 250 × 4.6 mm, 5 μm; eluent: *n*-hexane/*i*PrOH (99:1); flow: 1 mL min^−1^; detector: UV, 230 nm), yielding a fraction composed of methyl tri-*O*-benzoyl-α-d-arabinopyranoside (*t*_R_ = 24.3 min), which showed the same ECD spectrum as that reported in the literature [23].

### 3.5. Hydrolysis of Zeamide (***1***)

Zeamide (200 μg) was hydrolyzed by dissolving it in aqueous 1 M KOH (1.5 mL), and keeping the obtained solution for 72 h at 100 °C in a sealed tube. The resulting solution was partitioned between H_2_O/MeOH (8:2) and CHCl_3_, the organic layer was dried, dissolved in H_2_O/MeOH (8:1) and analyzed by LC-MS for sphingosines without any further purification. A 2.6-μ Kinetex C-18 column (150 × 2.1 mm) maintained at 25 °C was eluted at 200 μL min^−1^ with 0.1% HCOOH in H_2_O and MeOH. The gradient program was as follows: 60% MeOH 1 min, 60→100% MeOH over 40 min, 100% MeOH 16 min. Mass spectra were acquired in positive ion detection mode and the data were analyzed using the suite of programs Xcalibur.

The mixture was then benzoylated and purified by normal-phase HPLC as described above, yielding two fractions composed of, respectively, benzoylated sphingosines (*t*_R_ = 21.5) and benzoylated dihydrosphingosines (*t*_R_ = 16.7 min). Their ECD spectra matched those reported for, respectively, d-*erytro*-sphingosine and d-*erytro*-dihydrosphingosine [24].

## Figures and Tables

**Table 1:** NMR data of zeamide (**1**) (^1^H 700 MHz, ^13^C 175 MHz, CD_3_OD) *^a^*.

Position	δ_C_, Type *^b^*		δ_H_, mult ( *J* in Hz) *^c^*	HMBC *^d^*
1	65.7, CH_2_	a	4.24, ddd (10.9, 7.2, 4.2)	
		b	3.96, ddd (10.9, 6.4, 2.7)	
2	55.5, CH		3.91, ddd (9.0, 4.2, 2.7)	1‴
3	72.5, CH		4.10, t (8.3)	1, 2, 4, 5
4	131.2, CH		5.45, dd (15.3, 7.7)	3, 6
5	135.0, CH		5.71, ddd (15.3, 6.9, 6.9)	3, 6, 7
6	33.5, CH_2_		2.02, m	4, 5, 7
7	30.5, CH_2_		1.37, m	
1′	76.5, CH		4.01, ddd (9.9, 8.0, 2.7)	6′
2′	72.9, CH		4.27, t (2.7)	1′, 3′, 4′, 6′
3′	72.9, CH		3.36, dd (9.8, 2.7)	4′
4′	74.1, CH		3.63, t (9.5)	2′, 3′, 5′
5′	77.3, CH		3.41, t (9.3)	4′, 6′
6′	78.9, CH		3.91, t (9.6)	4′, 5′, 1″
1′′	101.5, CH		5.36, d (3.7)	6′, 3″, 5″
2″	71.1, CH		3.76, dd (9.9, 3.7)	
3″	70.8, CH		3.91, dd (9.9, 3.7)	
4″	71.1, CH		3.88, br. s	3″
5″	64.9, CH	a	4.36, br. d (12.7)	1″, 3″
		b	3.54, dd (12.7, 2.2)	1″, 3″
1‴	175.9, C			
2‴	37.4, CH_2_		2.21, t (7.6)	1‴, 3‴
3‴	27.2, CH_2_		1.58	1‴, 2‴

*^a^* NMR data of minor homologues with a saturated sphingosine. ^1^H signals: H-1a: 4.28, ddd (10.9, 7.9, 3.8); H-1b: 3.91, m; H-2: 3.84, ddd (9.0, 4.2, 2.7); H-3: 3.67, br. t (8.0); H-4a: 1.56, m; H-4b: 1.35, m. ^13^C signals: C-1: 65.9, CH_2_; C-2: 55.8, CH; C-3: 70.7, CH; C-4: 34.6, CH_2_; *^b^* Additional ^13^C signals: 40.4, CH_2_, *iso*-chain ω-2; 32.2, CH_2_, *n*-chain ω-2; 29.3, CH, *iso*-chain ω-1; 23.8, CH_2_, *n*-chain ω-1; 23.2, CH_3_, *iso*-chain ω; 20.3, CH_3_, internal branch; 14.5, CH_3_, *n*-chain ω; ppm; *^c^* Additional ^1^H signals: 1.53, nonet (6.6), *iso*-chain ω-1; 1.29, broad, alkyl chain protons; 1.18, m, *iso*-chain ω-2; 0.90, t (7.1), *n*-chain ω; 0.88, d (6.7), *iso*-chain ω; 0.86, d (6.8), internal branch methyl protons; *^d^* HMBC correlations from proton stated to the indicated carbon.

**Table 2:** Fatty acyl composition of zeamide (**1**).

Fatty Acid Methyl Ester	
methyl 13-methyltetradecanoate ( *iso*-C_15_)	22.6%
methyl 14-methylpentadecanoate ( *iso*-C_16_)	21.0%
methyl hexadecanoate ( *n*-C_16_)	25.4%
methyl 10-methylhexadecanoate (10Me-C_17_)	12.9%
methyl 15-methylhexadecanoate ( *iso*-C_17_)	14.2%
methyl octadecanoate ( *n*-C_18_)	3.9%

**Table 3:** Sphingosine composition of zeamide (**1**).

**Sphingosine**	
(2 *S*,3*R*,4*E*)-2-amino-4-tetracosene-1,3-diol (C_24_ d-*erythro*-sphingosine, d24:1)	3.5%
(2 *S*,3*R*,4*E*)-2-amino-4-pentacosene-1,3-diol (C_25_ d-*erythro*-sphingosine, d25:1)	2.7%
(2 *S*,3*R*,4*E*)-2-amino-4-hexacosene-1,3-diol (C_26_ d-*erythro*-sphingosine, d26:1)	62.1%
(2 *S*,3*R*,4*E*)-X-methyl-2-amino-4-hexacosene-1,3-diol (branched d27:1)	3.4%
(2 *S*,3*R*,4*E*)-2-amino-4-heptacosene-1,3-diol (C_27_ d-*erythro*-sphingosine, d27:1)	4.5%
(2 *S*,3*R*,4*E*)-2-amino-4-octacosene-1,3-diol (C_28_ d-*erythro*-sphingosine, d28:1)	5.2%
**Total unsaturated sphingosines**	**81.4%**
(2 *S*,3*R*)-2-amino-1,3-tetracosenediol (C_24_ d-*erythro*-dihydrosphingosine, d24:0)	1.8%
(2 *S*,3*R*)-X-methyl-2-amino-1,3-tetracosenediol (branched d25:0)	1.1%
(2 *S*,3*R*)-2-amino-1,3-pentacosenediol (C_25_ d-*erythro*-dihydrosphingosine, d25:0)	1.3%
(2 *S*,3*R*)-2-amino-1,3-hexacosenediol (C_26_ d-*erythro*-dihydrosphingosine, d26:0)	11.4%
(2 *S*,3*R*)-2-amino-1,3-heptacosenediol (C_27_ d-*erythro*-dihydrosphingosine, d27:0)	1.8%
(2 *S*,3*R*)-2-amino-1,3-octacosenediol (C_28_ d-*erythro*-dihydrosphingosine, d28:0)	1.2%
**Total saturated sphingosines**	**18.6%**

## Supplementary Materials

Supplementary Materials are available online. Figure S1: Structures of some unusual glycolipids from sponges; Figure S2: Sections of the *z*-TOCSY spectrum of zeamide (**1**); Figure S3: LC-HRMS analysis for sphingosines of the basic hydrolysis product of zeamide (**1**), and copies of the MS and one- and two-dimensional NMR spectra of **1**.

## References

[1] Paulick M.G., Bertozzi C.R. (2008). The Glycosylphosphatidylinositol Anchor: A Complex Membrane-Anchoring Structure for Proteins. Biochemistry.

[2] Murata D., Nomura K.H., Dejima K., Mizuguchi S., Kawasaki N., Matsuishi-Nakajima Y., Ito S., Gengyo-Ando K., Kage-Nakadai E., Mitani S. (2012). GPI-anchor synthesis is indispensable for the germline development of the nematode *Caenorhabditis elegans*. Mol. Biol. Cell.

[3] Bundy M.G.R., Kosentka P.Z., Willet A.H., Zhang L., Miller E., Shpak E.D. (2016). A Mutation in the Catalytic Subunit of the Glycosylphosphatidylinositol Transamidase Disrupts Growth, Fertility, and Stomata Formation. Plant Physiol..

[4] Martin K.L., Smith T.K. (2006). Phosphatidylinositol synthesis is essential in bloodstream form *Trypanosoma brucei*. Biochem. J..

[B5-molecules-22-01455] 5.It must be noted in this respect that GIPCs are quite difficult to study because of their unfavorable solubility properties, and generally require specific extraction procedures (see also ref. [10].). Therefore, it is likely that out present knowledge on the distribution of GIPCs in living organisms is biased by the methods used for their isolation and the interests of the researchers performing the studies.

[6] Gronnier J., Germain V., Gouguet P., Cacas J.-L., Mongrand S. (2016). GIPC: Glycosyl Inositol Phospho Ceramides, the major sphingolipids on earth. Plant Signal. Behav..

[7] Mayor S., Riezman H. (2004). Sorting GPI-anchored proteins. Nat. Rev. Mol. Cell Biol..

[8] Ferguson M.A.J., Kinoshita T., Hart G.W., Varki A., Cummings R.D., Esko J.D. (2009). Glycosyl Phosphatidylinositol Anchors. Essentials of Glycobiology.

[9] Guimarães L.L., Toledo M.S., Ferreira F.A.S., Straus A.H., Takahashi H.K. (2014). Structural diversity and biological significance of glycosphingolipids in pathogenic and opportunistic fungi. Front. Cell. Infect. Microbiol..

[10] Cacas J.L., Buré C., Furt F., Maalouf L.P., Badoc A., Cluzet S., Schmitter J.M., Antajan E., Mongrand S. (2013). Biochemical survey of the polar head of plant glycosylinositolphosphoceramides unravels broad diversity. Phytochemistry.

[11] Hsieh T.C.Y., Kaul K., Laine R.A., Lester R.L. (1978). Structure of a major glycophosphoceramide from tobacco leaves, PSL-I: 2-deoxy-2-acetamido-d-glucopyranosyl(α1→4)-d-glucuronopyranosyl (α1→2)myoinositol-1-*O*-phosphoceramide. Biochemistry.

[12] Costantino V., Fattorusso E., Imperatore C., Mangoni A. (2008). J-Coupling Analysis for Stereochemical Assignments in Furanosides: Structure Elucidation of Vesparioside B, a Glycosphingolipid from the Marine Sponge Spheciospongia Vesparia. J. Org. Chem..

[13] Costantino V., Fattorusso E., Mangoni A., Di Rosa M., Ianaro A. (1997). Glycolipids from Sponges. 6. Plakoside A and B, Two Unique Prenylated Glycosphingolipids with Immunosuppressive Activity from the Marine Sponge Plakortis Simplex. J. Am. Chem. Soc..

[14] Costantino V., Fattorusso E., Imperatore C., Mangoni A. (2003). Ectyoceramide, the First Natural Hexofuranosylceramide from the Marine Sponge Ectyoplasia Ferox. Eur. J. Org. Chem..

[15] Costantino V., Fattorusso E., Imperatore C., Mangoni A. (2002). Isocrasserides, Novel Glycolipids with a Five-Membered Cyclitol Widely Distributed in Marine Sponges. J. Nat. Prod..

[16] Costantino V., Fattorusso E., Imperatore C., Mangoni A. (2001). Plaxyloside from the Marine Sponge Plakortis Simplex: An Improved Strategy for NMR Structural Studies of Carbohydrate Chains. Eur. J. Org. Chem..

[17] Costantino V., Fattorusso E., Mangoni A., Di Rosa M., Ianaro A. (1999). Glycolipids from Sponges. VII.1 Simplexides, Novel Immunosuppressive Glycolipids from the Caribbean Sponge Plakortis Simplex. Bioorg. Med. Chem. Lett..

[18] Barbieri L., Costantino V., Fattorusso E., Mangoni A. (2005). Glycolipids from Sponges. Part 16. 1 Discoside, a Rare *myo*-Inositol-Containing Glycolipid from the Caribbean Sponge Discodermia Dissoluta. J. Nat. Prod..

[19] Nigou J., Gilleron M., Puzo G. (2003). Lipoarabinomannans: From structure to biosynthesis. Biochimie.

[20] Costantino V., Della Sala G., Mangoni A., Perinu C., Teta R. (2012). Blurring the Boundary between Bio- and Geohopanoids: Plakohopanoid, a C 32 Biohopanoid Ester from Plakortis Cf. Lita. Eur. J. Org. Chem..

[21] Lamoral-Theys D., Fattorusso E., Mangoni A., Perinu C., Kiss R., Costantino V. (2011). Evaluation of the Antiproliferative Activity of Diterpene Isonitriles from the Sponge Pseudoaxinella Flava in Apoptosis-Sensitive and Apoptosis-Resistant Cancer Cell Lines. J. Nat. Prod..

[22] Costantino V., Fattorusso E., Mangoni A., Tringali C. (2001). Bioactive Compounds from Natural Sources.

[23] Costantino V., Fattorusso E., Imperatore C., Mangoni A. (2005). Vesparioside from the Marine Sponge Spheciospongia vesparia, the First Diglycosylceramide with a Pentose Sugar Residue. Eur. J. Org. Chem..

[24] Munesada K., Yuasa M., Suga T. (1991). Cerebrosides of frog brain. Structure of the ceramide part of the cerebrosides. J. Chem. Soc. Perkin Trans. 1.

[25] Lee O.O., Chui P.Y., Wong Y.H., Pawlik J.R., Qian P.-Y. (2009). Evidence for Vertical Transmission of Bacterial Symbionts from Adult to Embryo in the Caribbean Sponge *Svenzea zeai*. Appl. Environ. Microbiol..

[26] Costantino V., Fattorusso E., Imperatore C., Mangoni A., Teta R. (2009). Amphiceramide A and B, novel glycosphingolipids from the marine sponge *Amphimedon compressa*. Eur. J. Org. Chem..

[27] Wilson M.C., Mori T., Rückert C., Uria A.R., Helf M.J., Takada K., Gernert C., Steffens U.A.E., Heycke N., Schmitt S. (2014). An environmental bacterial taxon with a large and distinct metabolic repertoire. Nature.

[28] Hochmuth T., Niederkruger H., Gernert C., Siegl A., Taudien S., Platzer M., Crews P., Hentschel U., Piel J. (2010). Linking chemical and microbial diversity in marine sponges: Possible role for poribacteria as producers of methyl-branched fatty acids. ChemBioChem.

[29] Della Sala G., Hochmuth T., Teta R., Costantino V., Mangoni A. (2014). Polyketide synthases in the microbiome of the marine sponge *Plakortis halichondrioides*: A metagenomic update. Mar. Drugs.

