# Supplementary material for: Zeamide, a Glycosylinositol Phosphorylceramide with the Novel Core Arap(1β→6)Ins Motif from the Marine Sponge Svenzea zeai
Source: Molecules. 2017 Sep 1;22(9):1455. doi: 10.3390/molecules22091455 (PMC6151786; doi:10.3390/molecules22091455)
Supplement: Supplementary file 1 [file molecules-22-01455-s001.pdf]

Zeamide, a glycosylinositol phosphorylceramide with the novel core Ara*p* (1β→6)Ins motif from the marine sponge *Svenzea zeai*

Gerardo Della Sala,[†] *Roberta Teta,*[†] *Germana Esposito,*[†] *Joseph R. Pawlik,*[‡] *Alfonso Mangoni,*[†] *Valeria Costantino*[†,*]

[†] *The NeaNat Group, Dipartimento di Farmacia, Università degli Studi di Napoli Federico II, via D. Montesano 49, 80131 Napoli, Italy*
[‡] *Department of Biology and Marine Biology, University of North Carolina Wilmington, Center for Marine Science, 5600 Marvin K Moss Lane, Wilmington, NC 28409, USA.*

Supporting Information

Table of contents

Figure S1. Structures of some unusual glycopids from sponges S2
Figure S2. Sections of the *z*-TOCSY spectrum of zeamide (**1**) S3
Figure S3. LC-HRMS analysis for sphingosines of the basic hydrolysis product of zeamide (**1**) ... S4
High-resolution ESI mass spectrum of zeamide (**1**) S5
^{1}H-NMR spectrum of zeamide (**1**) (700 MHz, CD_3OD) - full spectral windows S6
^{1}H-NMR spectrum of zeamide (**1**) (700 MHz, CD_3OD) - expansion S7
COSY spectrum of zeamide (**1**) (700 MHz, CD_3OD) S8
COSY spectrum of zeamide (**1**) (700 MHz, CD_3OD) - expansion S9
HSQC spectrum of zeamide (**1**) (700 MHz, CD_3OD) S10
HSQC spectrum of zeamide (**1**) (700 MHz, CD_3OD) - expansion S11
HMBC spectrum of zeamide (**1**) (700 MHz, CD_3OD) S12
^{1}H-^{31}P HMBC spectrum of zeamide (**1**) (700 MHz, CD_3OD) S13

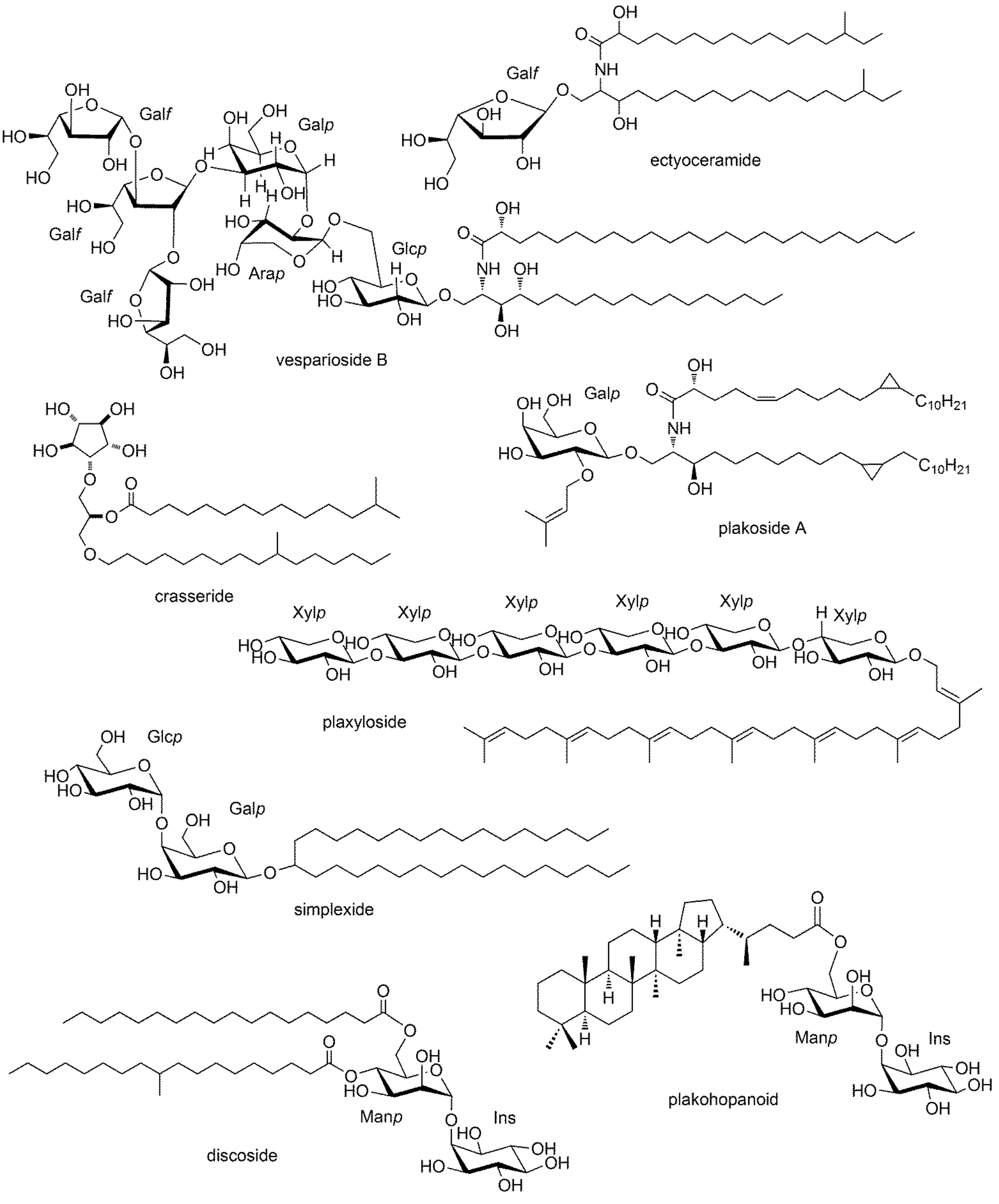

Figure S1. Structures of some unusual glycolipids from sponges cited in the main text. Many of these compounds have been isolated as mixture of homologues; one representative homologue is presented here.

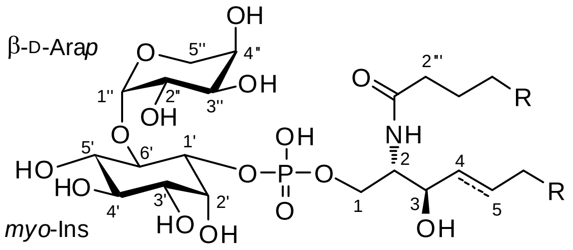

1

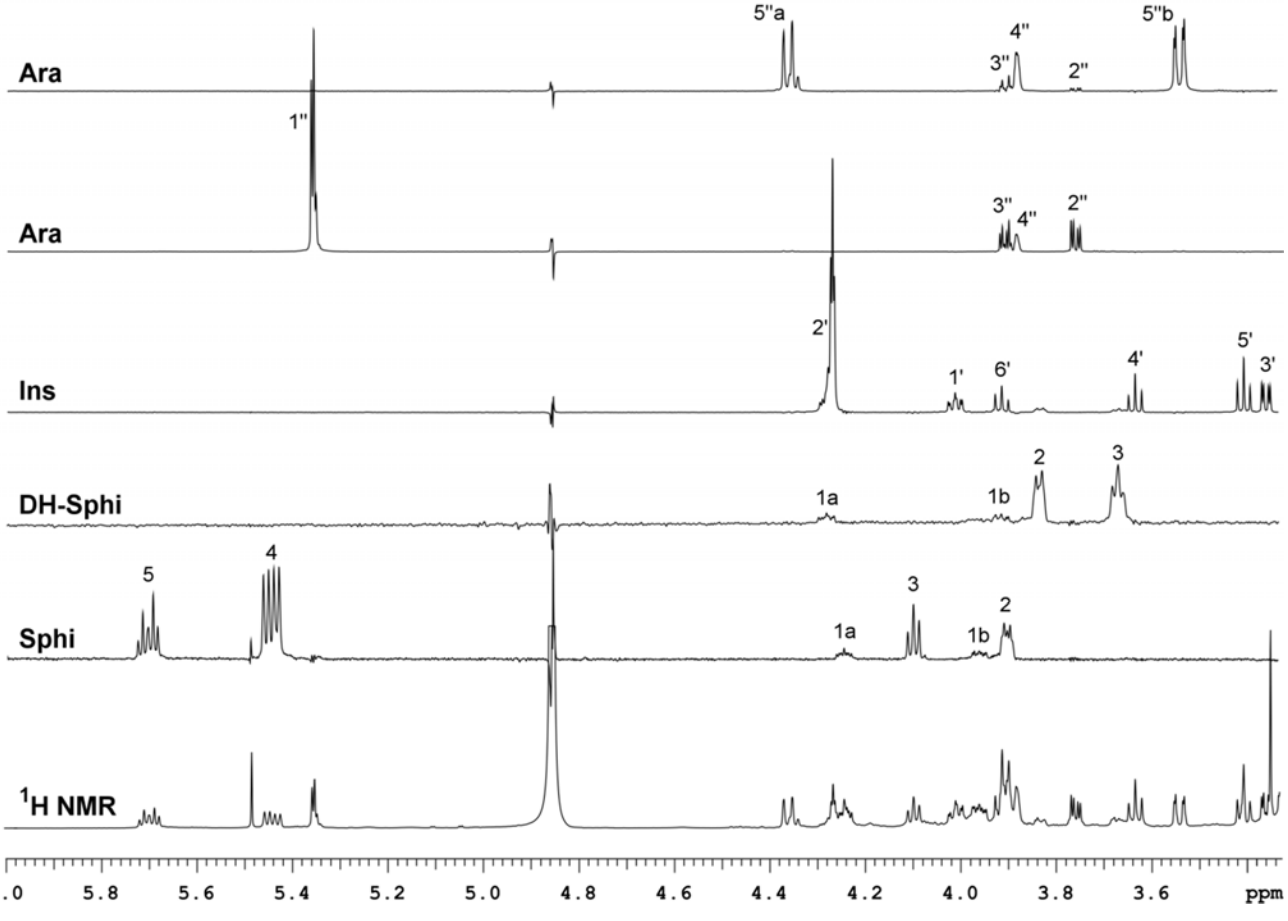

Figure S2. Sections of the 2D *z*-TOCSY spectrum (700 MHz, CD3OD, mixing time 100 ms) of zeamide (**1**), showing partial or complete subspectra of the spin-systems of the molecule.
From the bottom up: ^{1}H NMR spectrum; section at δ 5.45 (H-4), sphingosine protons; section at δ 1.56 (H-4b), dihydrosphingosine protons; section at δ 4.27 (H-2'), inositol protons; section at δ 5.36 (H-1''), arabinose protons; section at δ 3.54 (H-5''b), arabinose protons.

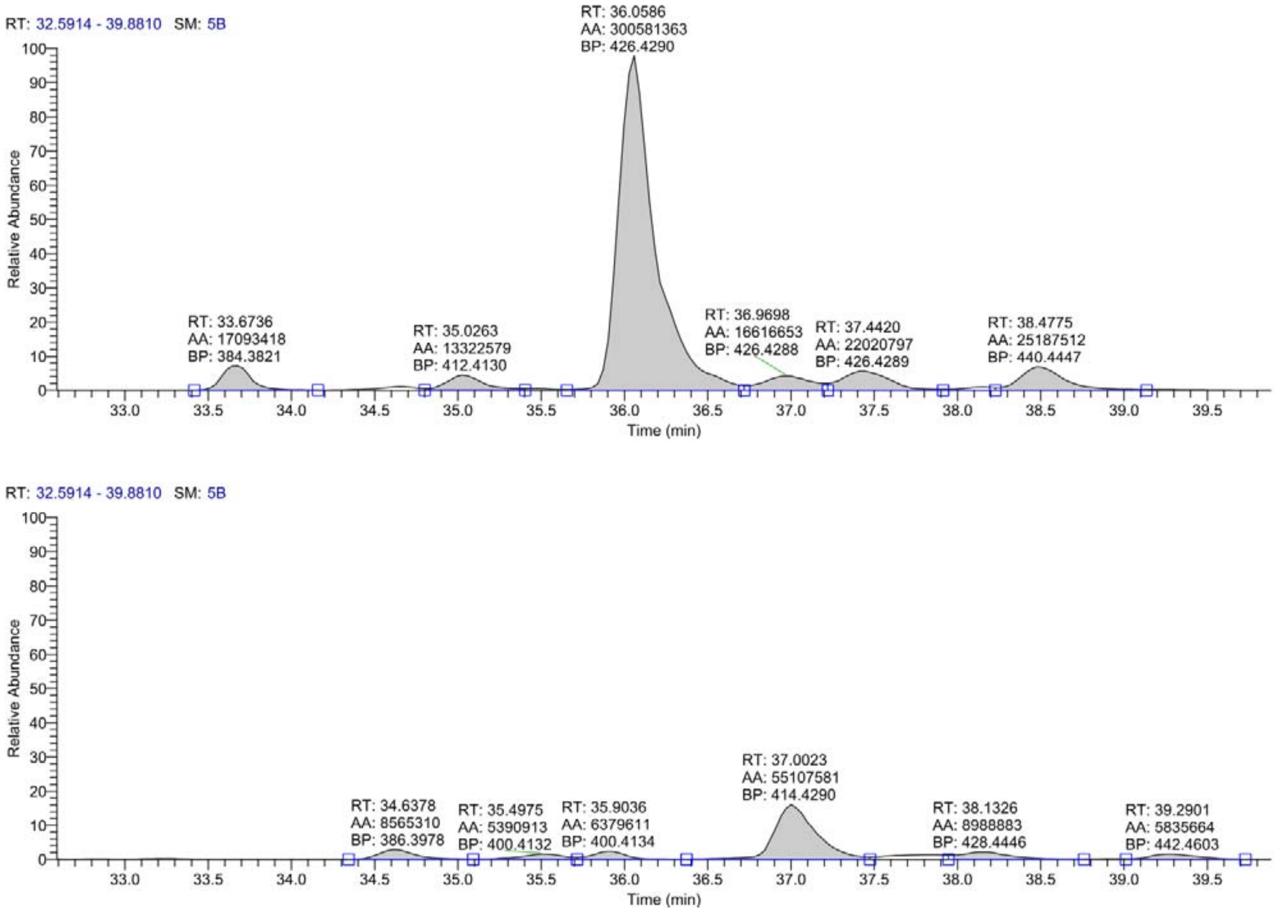

Figure S3. LC-HRMS analysis for sphingosines of the basic hydrolysis product of zeamide (**1**). Upper chromatogram: extracted-ion chromatogram at *m*/*z* 384.3834, 398.3990, 412.4146, 426.4302, 440.4458 (C_{24}-C_{28} sphingosines). Lower chromatogram: extracted-ion chromatogram at *m*/*z* 386.3990, 400.4146, 414.4302, 428.4458, 442.4614 (C_{24}-C_{28} dihydrosphingosines). Each chromatographic peak is labeled with retention time, area, and exact mass. The two cromatograms have the same vertical scale.

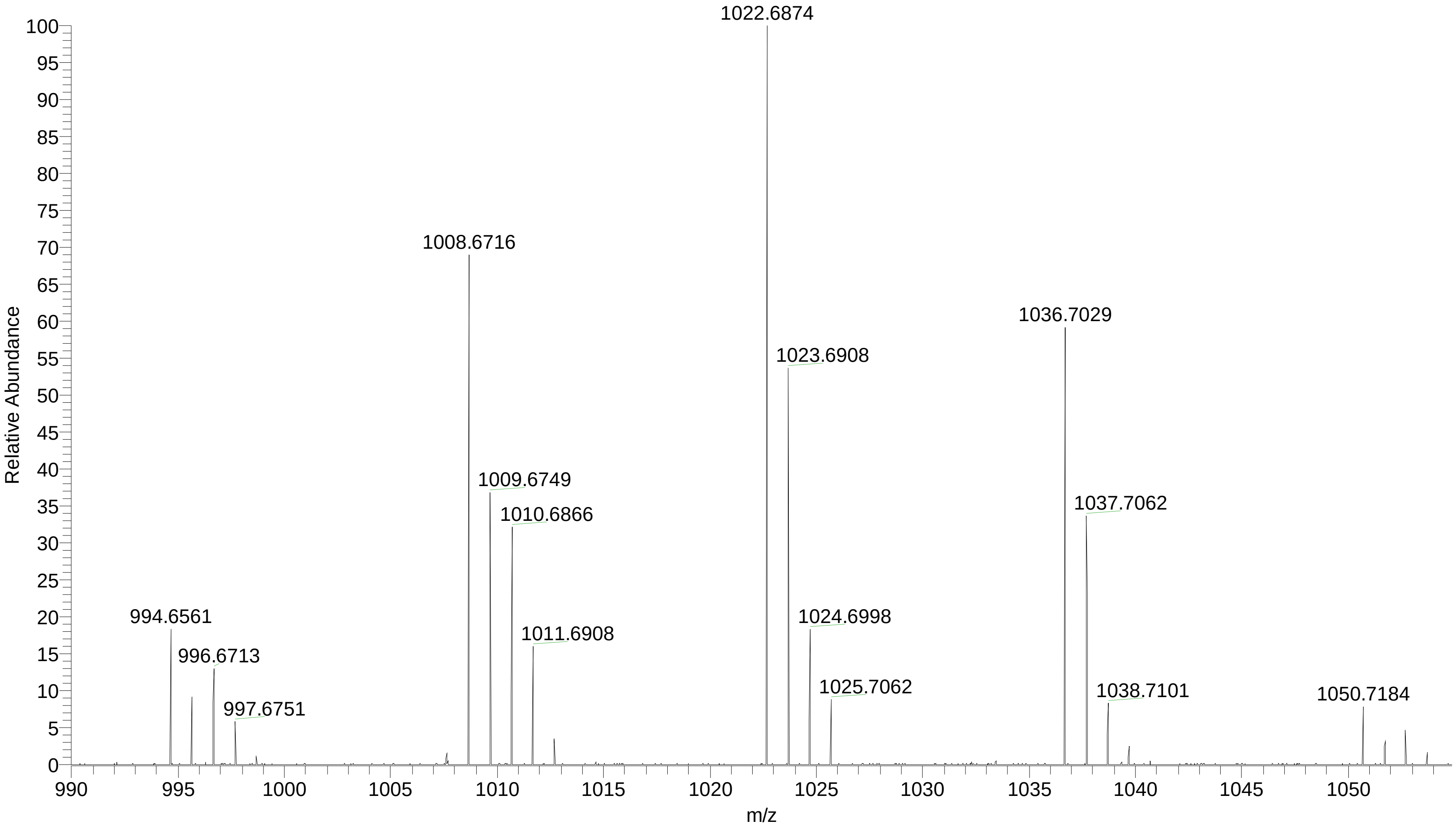

High-resolution ESI mass spectrum of zeamide (**1**)

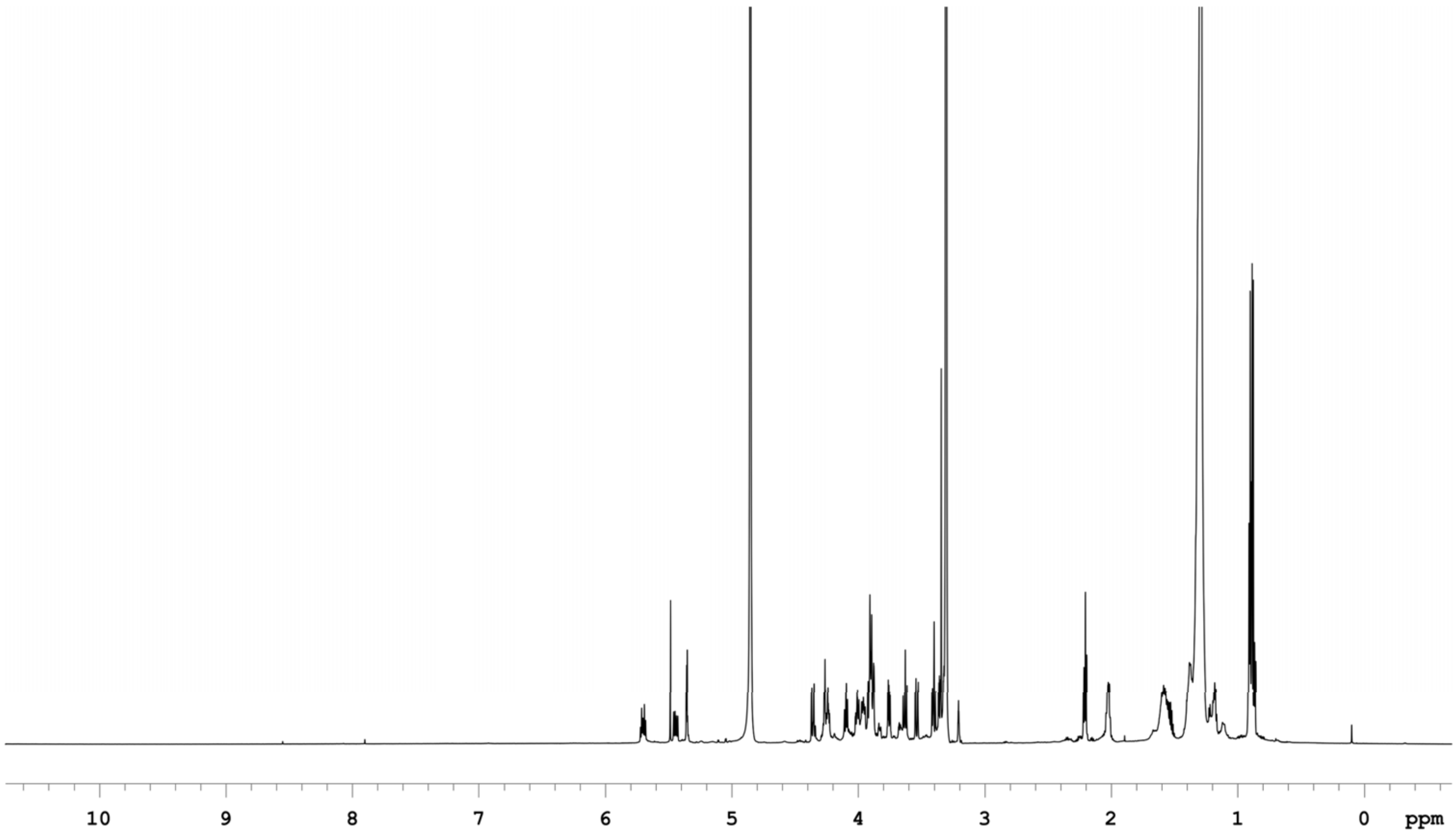

^{1}H-NMR spectrum of zeamide (**1**) (700 MHz, CD_3OD) – full spectral window

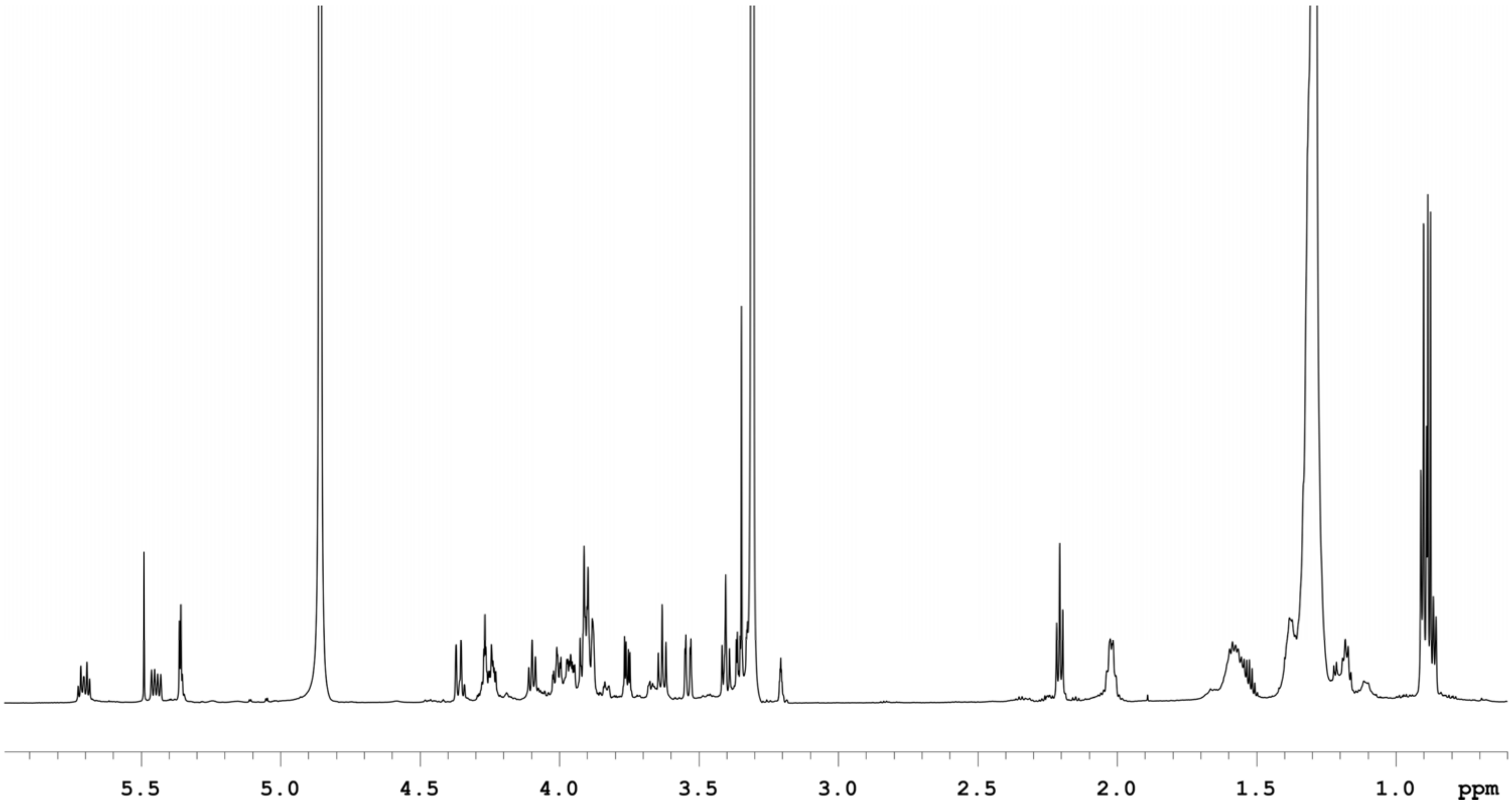

^{1}H-NMR spectrum of zeamide (**1**) (700 MHz, CD_3OD) – expansion

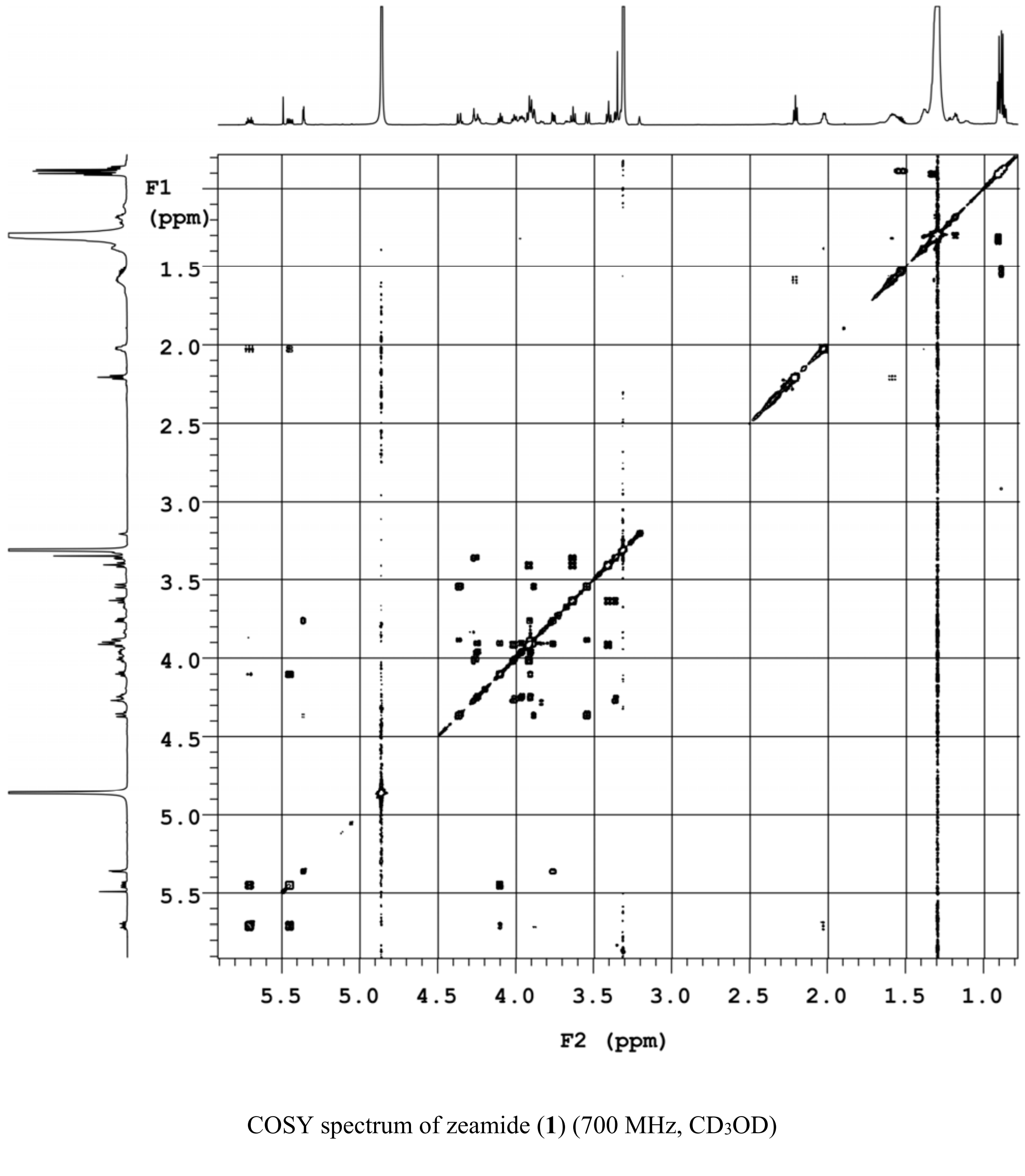

COSY spectrum of zeamide (**1**) (700 MHz, CD_3OD)

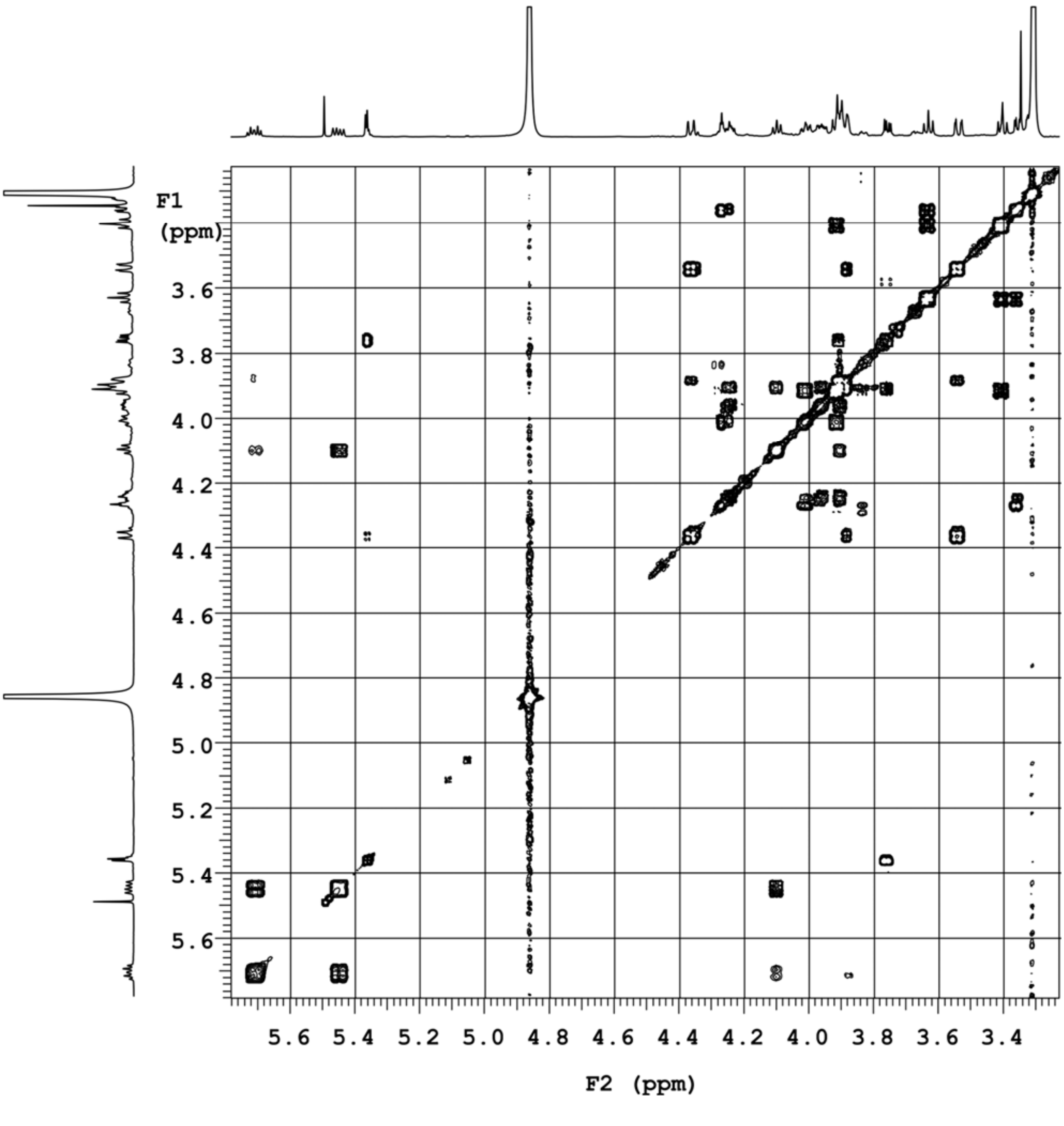

COSY spectrum of zeamide (**1**) (700 MHz, CD_3OD) – expansion

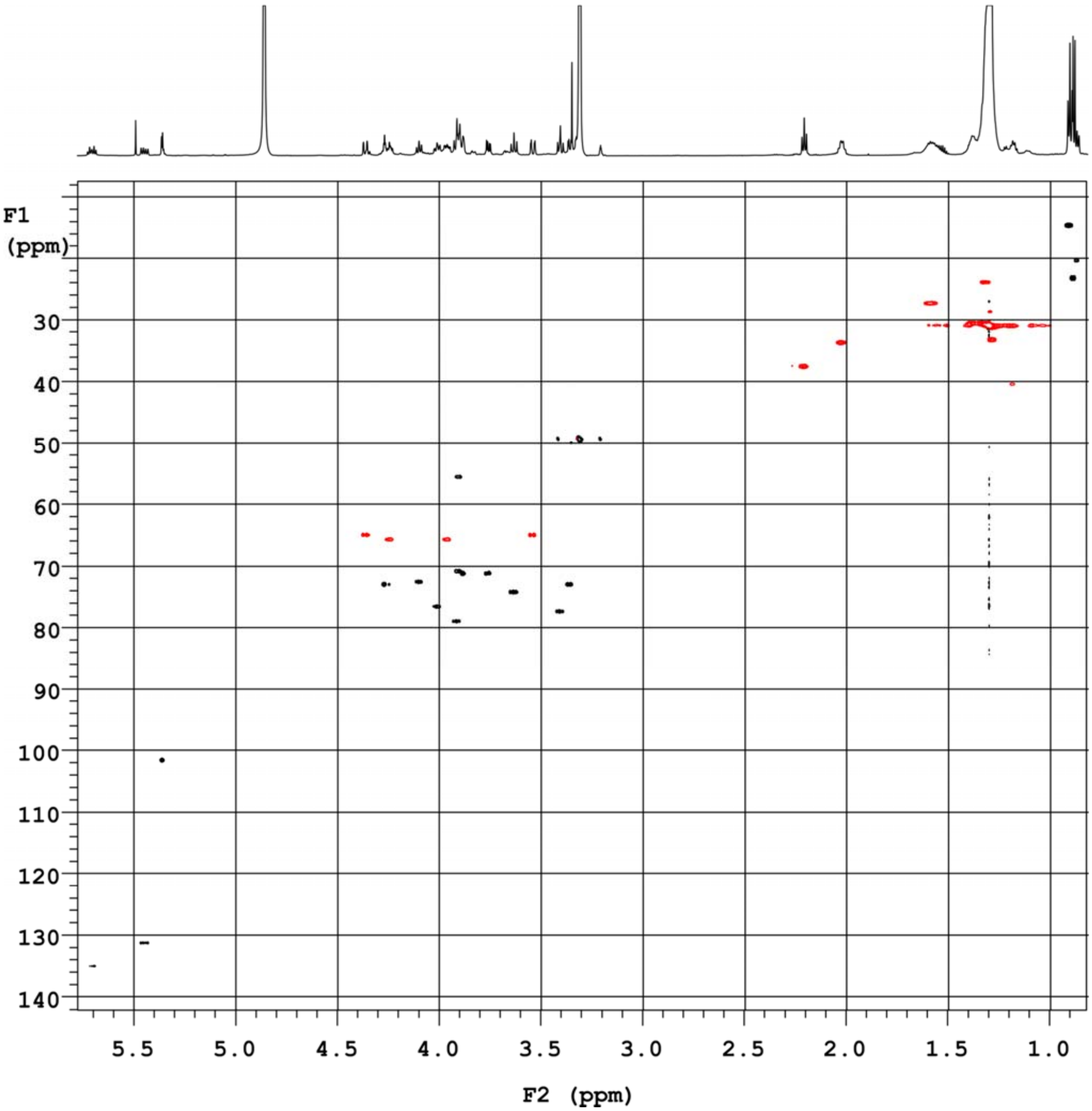

HSQC spectrum of zeamide (**1**) (700 MHz, CD_3OD)

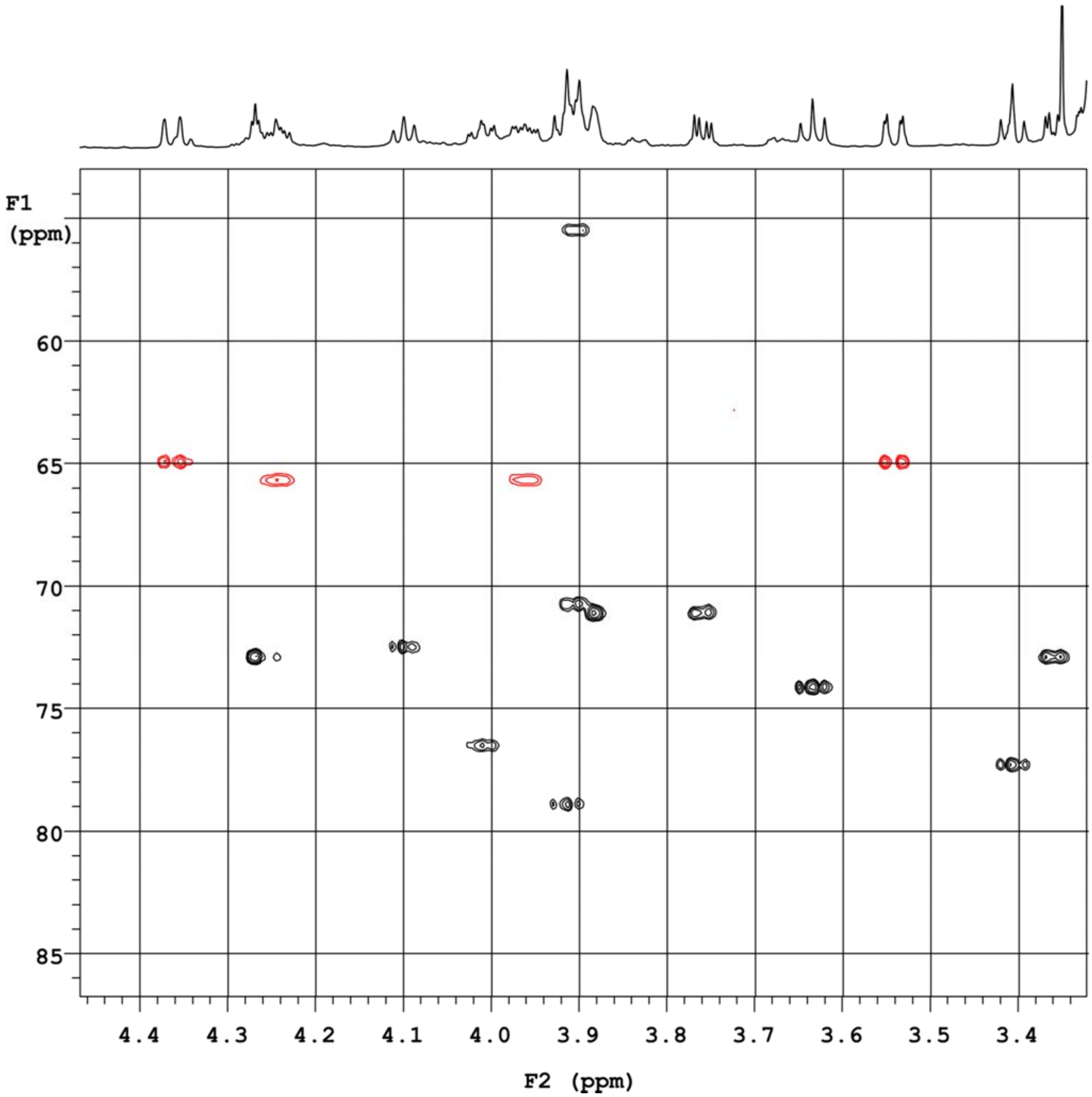

HSQC spectrum of zeamide (**1**) (700 MHz, CD_3OD) – expansion

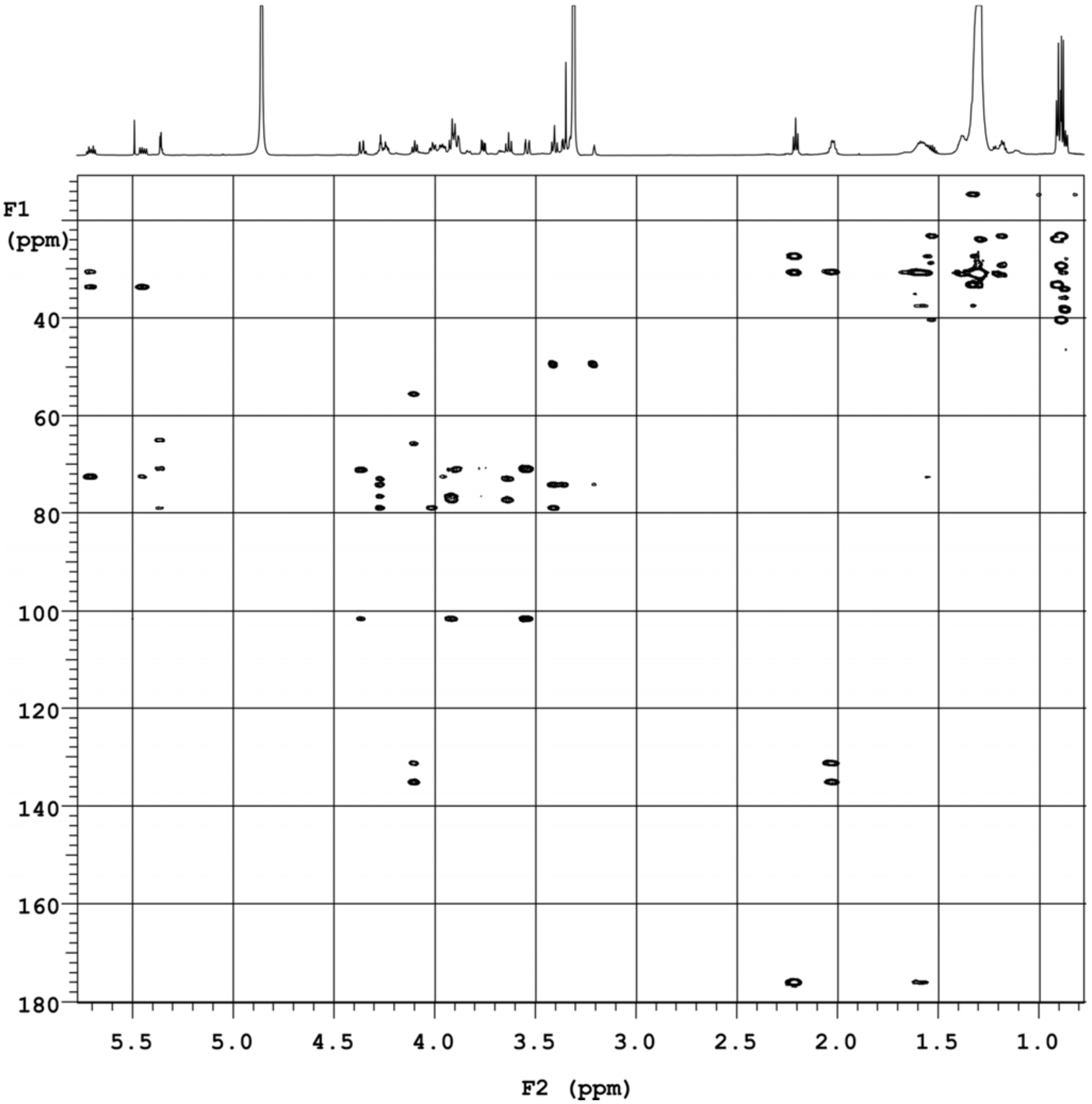

HMBC spectrum of zeamide (**1**) (700 MHz, CD_3OD)

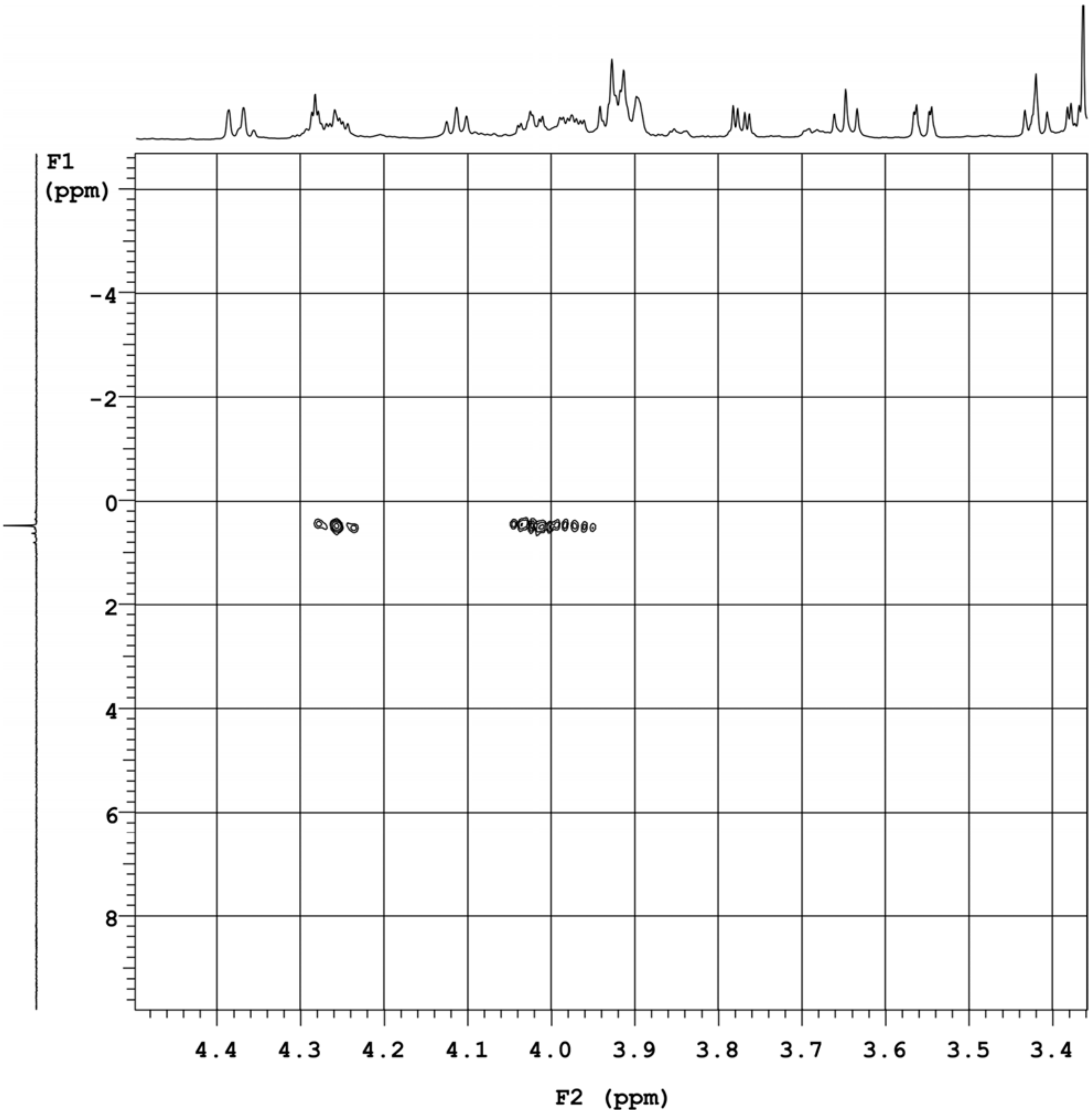

^{1}H-^{31}P HMBC spectrum of zeamide (**1**) (700 MHz, CD_3OD)
